# Texas Isolates Closely Related to *Bacillus anthracis* Ames

**DOI:** 10.3201/eid1409.080076

**Published:** 2008-09

**Authors:** Leo J. Kenefic, Talima Pearson, Richard T. Okinaka, Wai-Kwan Chung, Tamara Max, Matthew N. Van Ert, Chung K. Marston, Kathy Gutierrez, Amy K. Swinford, Alex R. Hoffmaster, Paul Keim

**Affiliations:** Northern Arizona University, Flagstaff, Arizona, USA (L.J. Kenefic, T. Pearson, R.T. Okinaka, W.-K. Chung, T. Max, M.N. Van Ert, P. Keim); Los Alamos National Laboratory, Los Alamos, New Mexico, USA (R.T. Okinaka); Centers for Disease Control and Prevention, Atlanta, Georgia, USA (C.K. Marston, A. R. Hoffmaster); Texas Veterinary Medical Diagnostic Laboratory, College Station, Texas, USA (K. Gutierrez, A.K. Swinford); Translational Genomics Research Institute, Phoenix, Arizona, USA (P. Keim)

**Keywords:** *Bacillus anthracis*, SNP, MLVA, VNTR, Ames strain, letter

**To the Editor:** Forensic and epidemiologic investigation of the 2001 bioterrorism-associated anthrax attacks used multiple-locus variable-number tandem-repeat analysis (MLVA) to identify the attack strain as Ames (*1*). Strain identity was essential for subsequent molecular epidemiologic and forensic investigations of this biocrime. To more easily identify this particular strain, comparative whole-genome sequencing (*2*) and phylogenetic analyses were used to identify single-nucleotide polymorphisms (SNPs) that seem highly specific for Ames strain identification (*3*). Because *Bacillus anthracis* is a recently emerged clonal pathogen, these SNPs represent highly evolutionarily stable markers (*4*) that are amenable to many rapid and cost-effective analytical techniques.

MLVA and the Ames-specific SNP assay indicate that the Ames strain has been isolated from nature only 1 time, in southern Texas, USA. Several lineages of *B. anthracis* (*5*) have been ecologically established in North America. The A.Br.009 clade is the most successful and widely dispersed in North America, but it is not closely related to the Ames strain (*5*), which is a member of the A.Br.001 clade. Although A.Br.001 is not as successful as A.Br.009, it appears to be ecologically well established in southern Texas. Analyses of outbreaks in this region from 1974 to 2000 found 190 culture-confirmed cases clustered mainly in 5 counties (*6*). A major epizootic in Texas in 2001 paralleled this trend. This outbreak (*6*) affected many deer species, horses, and bovids (total 1,637), which suggests that this clade is well established and not limited to cultivated areas and domesticated livestock. Previous molecular and epidemiologic analyses (*3*) of isolates from this region identified close, but not identical, matches to the Ames strain, which suggests that more intense surveillance in this region would likely yield more Ames and Ames-like isolates. Two recent (2006 and 2007) outbreaks in Texas confirmed this suggestion.

Isolates from the 2006 and 2007 outbreaks were initially screened by using an 8-marker MLVA system (MLVA8) as described by Keim et al. (*7*). The MLVA8 genotypes were identical to the *B. anthracis* Ames strain (GT62). Additional analysis by a 15-marker (MLVA15) variable-number tandem repeats (VNTR) system (*5*) again generated an MLVA15 genotype that was identical to the original Ames strain (A0462) and to the 2001 bioterrorism-associated attack strain (A2012) (Figure). Given the identical MLVA genotypes, could these natural strains be differentiated from the laboratory or biocrime Ames strain by using higher resolution genotyping?

**Figure:**
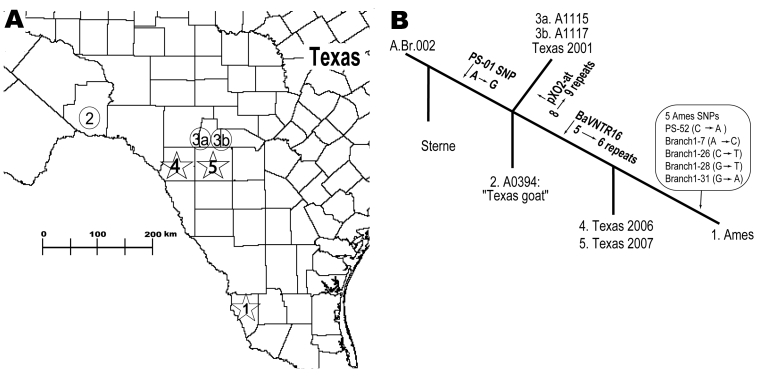
Geographic and phylogenetic relationships among strains closely related to *Bacillus anthracis* Ames strain. A) Spatial relationships among Ames-like isolates from southern Texas. 1, location of the original Ames strain, isolated from Jim Hogg County, Texas, in 1981; 2, closely related Texas 1997 goat isolate (A0394); 3a and 3b, Texas 2001 isolates; 4 and 5, most recent cases, i.e., Texas 2006 (Kinney County) and Texas 2007 (Uvalde County). B) Genetic relationships among isolates with variable-number tandem-repeat and single-nucleotide polymorphism (SNP) differences giving rise to that particular branch (arrows). The numbers at each branch terminus correlate with the numbers depicted on the map. SNP states are from ancestral to derived.

We developed 6 Ames strain–specific SNPs to address the potential that the Ames strain might reappear naturally and hinder epidemiologic and forensic investigations (*3*). We found that 5 of 6 SNP loci could be used to distinguish between all known natural isolates and laboratory or biocrime isolates (Figure). Results were consistent with our previous identification of a *B. anthracis* isolate from a goat kid in Texas in 1997 (A0394) as being closely related to the Ames strain (*3*). However, the 2006 and 2007 isolates from Texas were even more closely related to the Ames strain because they also shared the MLVA15 genotype with Ames. In contrast, the 1997 goat isolate differed by a single mutational step at the BaVNTR16 locus (Figure). Hence, 5 of 6 SNP markers enabled differentiation among Ames and Ames near relatives even when VNTR profiles were identical.

Resolution of nearly identical genotypes might also be accomplished by using additional VNTRs (*8*) or hypermutable loci (*9*). However, we doubt that this approach would be better than whole-genome sequencing with interrogation of resultant SNPs because these markers would most likely result in topologic conflicts due to homoplasy (*10*). The available epidemiologic data from other isolates included in this clade show that although the Ames clade is well established in southern Texas, no subsequently recovered natural isolates completely match the original Ames isolate.

The precision of subtyping assays is a matter of importance and debate for epidemiologic and, recently, forensic investigations. Strain identity is commonly used to infer a common source even when spatial and temporal data are not congruent. Moreover, the definition of a strain is somewhat unclear and relies on analytical methods that vary widely. Therefore, isolates may be erroneously excluded or included into a strain definition and disease outbreak as illustrated with the Ames strain and 2 contrasting approaches to identification. MLVA15 ties naturally occurring isolates to bioterrorism-associated attacks, while specific SNP assays can distinguish among them.

MLVA is an unbiased approach and can be used on any set of *B. anthracis* strains, although, as in the 2006 and 2007 Texas outbreaks, it can be limited in resolving power. In contrast, our SNP assays have great resolving power but are useful only for differentiating the Ames strain, thus limiting their value to categorical inclusion or exclusion in outbreaks. Future rational use of a battery of different molecular signatures will yield far greater insights into strain identity than the application of 1 specific signature.
