# Erratum—Vol. 14, No. 9

**DOI:** 10.3201/eid1410.071196c

**Published:** 2008-11

In Texas Isolates Closely Related to *Bacillus anthracis* Ames (L.J. Kenefic et al.), 3 author names were inadvertently omitted from the submitted article. They are Carla P. Trim, James M. Schupp, and Jodi Beaudry; each is from Northern Arizona University, Flagstaff, Arizona, USA. The complete author list as it should have appeared on the article: Leo J. Kenefic, Talima Pearson, Richard T. Okinaka, Wai-Kwan Chung, Tamara Max, Carla P. Trim, Jodi A. Beaudry, James M. Schupp, Matthew N. Van Ert, Chung K. Marston, Kathy Gutierrez, Amy K. Swinford, Alex R. Hoffmaster, and Paul Keim. The corrected article is available online from www.cdc.gov/EID/content/14/9/1494.htm.

